# Understanding sarcoma drug resistance one cell at a time

**DOI:** 10.20517/cdr.2021.87

**Published:** 2022-01-19

**Authors:** Salah-Eddine Cherradi-Lamhamedi, Danh Truong, Joseph A. Ludwig

**Affiliations:** Department of Sarcoma Medical Oncology, Division of Cancer Medicine, The University of Texas, MD Anderson Cancer Center, Houston, TX 77030, USA.

As true for any cancer, intrinsic and acquired drug resistance represent perhaps the single biggest obstacle limiting our ability to cure patients with metastatic disease. Yet, tackling this challenge has been particularly difficult for sarcoma. First and foremost, sarcomas are rare and diverse, which splits a class of tumors representing less than 1 percent of all malignancies into more than fifty smaller molecularly and histologically distinct entities, each having its own etiology, prognosis, and clinical presentation. Even the most “common” sarcoma subtypes (e.g., liposarcoma, leiomyosarcoma, or gastrointestinal stromal tumors) have annual incidences in the thousands in the United States, whereas rarer subtypes like desmoplastic small round cell tumors number less than fifty per year. Due to their scarcity, it’s not uncommon for just a few cell lines, organoids, or patient-derived tumor explants to exist for each sarcoma subtype. This shortage limits their usefulness to model the wide variety of innate and adaptive mechanisms that exist in human tumors. Many times, no representative preclinical model exists. The scarcity of FDA-approved drugs aimed at sarcoma-specific targets, often fusion proteins and transcription factors, is also problematic.

Complicating the ability to understand tumor drug resistance, many sarcomas exhibit pronounced intra-tumoral heterogeneity, manifested by heterogeneous cell populations of varied lineages and states of differentiation. It is not unusual for osteosarcoma, for example, to harbor cells with varying degrees of chaotic osteogenic, adipogenic, or chondrogenic cell fates. Similarly, some liposarcomas are classified into well-differentiated and dedifferentiated variants by their predominant differentiation status but usually harbor a blend of both^[1]^. Even tumors like Ewing sarcoma, which appear relatively homogeneous by light microscopy, can be subcategorized by the pathognomonic genomic translocation expressed and by the presence or absence of well-described point mutations in *STAG2*, *p53*, or *CDKN2A*^[2]^. Thus, while light microscopy and careful review by an experienced sarcoma pathologist remain the cornerstone of sarcoma diagnosis, a clear trend in the research setting is to deeply interrogate each tumor and precisely catalog its cellular constituencies using a wide array of genomic, proteomic, and other “-omic” technologies. As a direct result of this unparalleled opportunity to parse out distinct groups of malignant cells, a more nuanced view of sarcoma drug resistance must consider not only the global factors linked to each sarcoma subtype but also the intratumoral features such as physical and mechanical cues (e.g., extracellular matrix, stiffness or confined adhesiveness) that allow for clonal evolution in response to the selective pressures triggered by standard chemotherapy or biologically targeted therapy.

This has been achieved in other cancer types by engaging powerful single-cell technologies - before and after drug exposure - to determine a tumor’s baseline transcriptomic state and nascent cellular changes associated with drug resistance^[3-6]^. Single-cell and single-nucleus RNA-sequencing (sc/snRNA-seq) are the most common methods used currently and, when paired with freely available custom-made bioinformatic tools written in R or Python, provide the revolutionary ability to deeply interrogate intra-tumoral complexity^[7-9]^. Furthermore, while still in their infancy, single-cell proteomic technologies have introduced promising technologies that enable high-resolution spatial image - omic (SIO) analyses of human primary tumors, which allows one to learn not only *what* cell types exist but also *how* they interact in 2D or 3D space^[10]^. Though the clinical value of checkpoint blockade and cellular immunotherapies remains to be proven across the range of sarcoma subtypes, the advent of SIO, image mass cytometry, multi-color fluorescent microscopy, and other techniques should provide a fresh opportunity to examine whether such therapies can invoke an anti-cancer response. Complementing the trained eye of an experienced sarcoma pathologist, deep-learning and artificial intelligence, while still under-utilized, are primed to aid pathologists to make sense of the torrent of data generated from each slide and may allow them to perceive subtle cell-cell interactions that contribute to drug resistance and the feedback mechanisms that sustain them^[11]^.

Though classic measures of drug efficacy that rely on tumor size or patient survival will persist as important clinical study endpoints, a deeper appreciation of drug sensitivity/resistance must inevitably decipher how each patient’s tumor - and the cells within them - evade therapy. Like no time in history, the tools exist today to help cure cancer one cell at a time.

## DECLARATIONS

### Copyright

© The Author(s) 2022.
